# Extended Reality for Enhanced Telehealth During and Beyond COVID-19: Viewpoint

**DOI:** 10.2196/26520

**Published:** 2021-07-26

**Authors:** Triton Ong, Hattie Wilczewski, Samantha R Paige, Hiral Soni, Brandon M Welch, Brian E Bunnell

**Affiliations:** 1 Doxy.me, LLC Rochester, NY United States; 2 Biomedical Informatics Center Medical University of South Carolina Charleston, SC United States; 3 Department of Psychiatry University of South Florida Tampa, FL United States

**Keywords:** extended reality, virtual reality, augmented reality, mixed reality, telehealth, telemedicine, COVID-19, telepresence

## Abstract

The COVID-19 pandemic caused widespread challenges and revealed vulnerabilities across global health care systems. In response, many health care providers turned to telehealth solutions, which have been widely embraced and are likely to become standard for modern care. Immersive extended reality (XR) technologies have the potential to enhance telehealth with greater acceptability, engagement, and presence. However, numerous technical, logistic, and clinical barriers remain to the incorporation of XR technology into telehealth practice. COVID-19 may accelerate the union of XR and telehealth as researchers explore novel solutions to close social distances. In this viewpoint, we highlight research demonstrations of XR telehealth during the COVID-19 pandemic and discuss future directions to make XR the next evolution of remote health care.

## Introduction

In-person health care became limited during the COVID-19 pandemic. Social distancing, travel restrictions, and lockdowns forced many to perform and receive health care remotely via telecommunication (ie, telehealth) [1]. Telehealth emerged as a widely effective and accepted solution to support continuity of care throughout the ongoing pandemic [2-4]. Consensus among providers, patients, and policymakers indicates that the shift to telehealth will likely continue even after the pandemic ends [5-7]. To maintain current uptake and support delivery of the best possible care in the future, telehealth needs to supplement and transcend traditional models of in-person care.

As the health care industry adapts to telehealth, aspects of in-person treatment must be optimized for remote care (eg, conversational flow, physical evaluation, therapeutic presence). Some patients report reluctance to self-advocate during typical telehealth sessions because of poor eye contact and audio interference if more than one person speaks at a time [8]. Providers may find it difficult to build rapport and express empathy with patients via telehealth due to limited visibility of body language and the unavailability of physical presence [9]. Both providers and patients can encounter distractions as telehealth is conducted from within their homes [10]. Although telehealth appears set to become the new norm, novel approaches are needed to optimize patient outcomes by restoring critical aspects of in-person health care for remote formats and expanding clinical options to deliver health services at a distance.

Technologies that evoke presence—the perception, feeling, and interaction with simulations as if they were real [11]—can meaningfully impact the practice and outcomes of telehealth. Immersion into fully simulated virtual reality (VR) [12], simulated objects or overlays superimposed onto users’ real sight in augmented reality (AR) [13], or direct interaction between simulations and the physical world in mixed reality (MR) [14] each afford new ways to support, extend, and enhance health care practice in the shift to remote delivery. These VR, AR, and MR technologies—collectively referred to as extended reality (XR)—have been demonstrated for inpatient and outpatient psychiatric, medical, and rehabilitative applications with equal or greater effectiveness than their non-XR standard treatments [13,15-17]. However, research on XR as an extension of remote health care is comparatively recent and has yet to be synthesized.

The need to explore XR for telehealth has never been greater than in the fallout of COVID-19. Postponement of regular and preventive medical care, the psychological and developmental impacts of the extended pandemic, and escalating reports of provider burnout are harrowing signs on the health care horizon [18-26]. Research on the combination of XR and telehealth embodies recent events, addresses current limitations of conventional telehealth, and paves the path for the health care of tomorrow. We believe that XR telehealth research performed since the onset of COVID-19 will set the tone for research and innovations in the coming years. In this viewpoint, we provide a narrative review of current XR telehealth research and highlight future directions to address remaining barriers.

## XR for Telehealth Before COVID-19

The potential for reality-altering technologies in remote health care has been heralded since the earliest days of VR. Early VR required complex and costly computing hardware that kept the technology localized, for use by a single individual, and prohibitively expensive until the proliferation of the internet and affordable computing hardware [27,28]. Once the internet became widely available in the mid-1990s, surgical applications of VR and AR expanded to include multiuser supervision by remote experts, and detailed simulations to plan and practice surgical procedures [29-31]. Growing interest in interactive therapy drove MR technologies for at-home telerehabilitation, sometimes using off-the-shelf video game console hardware [32,33]. XR gradually matured with consumer-oriented hardware and software packages that led to interdisciplinary developments such as online VR for psychiatric treatments and sophisticated medical training simulations [34,35]. However, costs and technical complexity remained barriers to the wide deployment of XR for telehealth [36].

Trends in home computing and entertainment made user-friendly, robust, and polished XR equipment available for personal ownership in the 2010s, which facilitated the rapid growth in XR telehealth research and development [37]. Since then, XR telehealth has expanded to a wide variety of remote health care applications in AR telesurgery and telesupervision, MR training and simulations, VR telemental health, and telemonitoring of fully remote interventions and specialized medical equipment with VR/AR [13,38,39]. Similar growth occurred in consumer markets with reported use of entertainment VR apps and video games for therapeutic purposes such as mental wellness, identity exploration, healthy aging, and social anxiety [40-42].

Advances in internet infrastructure, computer technology, and portable consumer entertainment gradually decreased costs and increased consumer interest in XR devices. Increasing commercial availability of these devices and continued innovative research placed XR telehealth on a mainstream trajectory in 2019 [43].

## XR for Telehealth During COVID-19

### Resource Constraints and the Rise of Telehealth

In early 2020, the World Health Organization declared the COVID-19 pandemic and promoted social distancing to limit the spread [44]. Soon after, mass consumption of personal protective equipment (PPE) such as N95 face masks, medical-grade sanitizers, and disposable gloves led to global supply shortages for both health care workers and the general population [45]. The health care industry’s primary response to COVID-19 and PPE shortages was a rapid and widespread shift to remote services [4,46]. By June 2020, in-person health care visits were down by 30% while telehealth visits increased by up to 2013% [47]. This shift was even more pronounced for mental health services, which saw 70% fewer patients in person while telehealth sessions increased by up to 6558% [48,49].

XR telehealth was reported to be an effective solution that enhanced safety and reduced PPE consumption in COVID-19 health care settings. Two frontline case studies showed how medical specialists, unable to travel during quarantine, used AR to provide remote consultation and ventilator management for COVID-positive patients [50,51]. Intelligent AR information displays enhanced the workflows of frontline hospital staff to increase clinical efficiency, improve remote team communications, reduce COVID exposure by 51.5%, and decrease PPE consumption by 83% [52]. VR simulation systems for trauma and emergency medicine offered effective and high-fidelity alternatives to traditional supervision with less need to consume PPE for on-site medical training [53]. XR telehealth alternatives were also demonstrated for patient therapy. A VR group-singing intervention as respiratory therapy for spinal cord injury was found to be feasible, acceptable, enjoyable, and reported as less socially inhibited than the in-person prospects of the same intervention [54]. General population users in another feasibility study favored telemental health in VR over the standard webcam format [55]. Multiuser VR in analog telehealth conditions was found to be an ideal environment to conduct evidence-based cognitive behavioral therapy (CBT) in a space that felt immersive, expressive, private, anonymous, and free from judgment [56]. Overall, this published research shows that XR technologies complemented telehealth solutions to support frontline health care workers and maintain social distancing for critical evidence-based treatments during COVID-19.

### Access to Medical and Mental Health Care

In addition to social distancing, local and state governments imposed travel restrictions to limit the transmission of COVID-19 and reduce strain on health care systems [57]. However, these restrictions entailed collateral costs to the public’s health. Reduced public transportation disproportionately impacted lower-income and ethnic groups in urban areas, and further destabilized access to health care in rural regions with few local specialists [58-60]. Extended pandemic conditions also increased the global risk of psychological distress, impacted people’s daily habits and life plans, and subjected vulnerable populations to prolonged social isolation [57,61].

Barriers to health care access intensified during the pandemic and XR telehealth emerged as a responsive option. XR telehealth had a particularly significant impact to increase the immediacy of care and access among medically and geographically isolated populations who required continual rehabilitation services [62,63]. In addition to providing immersive and accessible care, XR telehealth connected people in virtual spaces to combat social isolation and maintain health-promoting social relationships over distances [64]. For example, location-based AR video games provided a protective effect for social, physical, and mental health during the pandemic [65-69]. Experts also promoted XR telehealth developments as a potential solution to address the downstream developmental impacts of the prolonged pandemic upon children and adolescents who eagerly take to new technologies [25,70].

### Burnout and Contagion Exposure Among Health Care Workers

The psychological distress of COVID-19 was particularly burdensome for health care workers. Sudden increases in workload, overcrowding, medical supply shortages, exposure to the virus, and the suffering of patients led to extreme physical and emotional strain upon frontline and hospital staff [22,71-73]. Health care workers were seven times more likely to exhibit severe COVID-19 symptoms than other workers due to their frequent and extreme exposure to contagion environments [74]. It is estimated that more than 3500 US frontline health care workers have died from COVID-19 contracted during their health care service [75]. Burnout among health care workers proved to be another contagion that spread within hospital wards with cascading staff turnover, compassion fatigue, and secondary traumatic stress [76,77]. Experts anticipate severe downstream impacts upon health care workers, and urge for evidence-based therapeutic interventions responsive to the impacts of COVID-19 and for methods to reduce health care workers’ exposure to the virus [78-81].

CBT for mindfulness is known to alleviate burnout and improve overall mental wellness among health care workers [71,82-84]. XR virtual visits have emerged as a promising technology for stress reduction using CBT and other evidence-based telemental health approaches [85,86]. With the advent of telehealth, VR and AR for COVID-related stress and trauma therapies have been promoted for distribution among frontline health care workers [51,87,88]. Although these studies are ongoing, VR telehealth for posttraumatic stress among survivors of COVID-19 has shown promising effects [89,90], and it is reasonable to expect these effects to generalize to providers from these same environments and traumatic experiences [91]. In addition to targeting burnout among health care workers, XR has been used to innovate health care workflows with remote, intelligent, and burnout-reducing solutions. A preliminary application of AR telesurgical consultations allowed remote specialists to provide real-time expertise for COVID-positive patients without travel or exposure risks, paving a way for large-scale future implementation [50]. Interconnected AR systems improved infection control, increased access to specialist remote supervision, reduced time spent in contagion environments, and enhanced clinical workflows in frontline health care environments [51,52].

### Economic and Professional Pressures on Health Care Providers

The accumulated effects of social distancing, chronic resource shortages, travel restrictions, hospital surges, and pandemic stress created instability for current and future health care providers. By August 2020, more than 16,000 private practices had permanently closed with 41% of their peers facing the same fate with unsustainable loss of staff, patients, and income [20]. The subsequent low viability of for-profit clinics exacerbated concerns with reduced health care support [92]. This new fragility in health care networks was particularly straining for already underserved rural regions and ethnic populations [93,94]. Prolonged pandemic conditions further inhibited traditional pathways to hands-on health care experience and clinical supervision, which delayed professional development of the next generation of health care providers [95-97]. Governments, hospitals, and health care providers rallied to support public health during COVID-19, but extended pandemic conditions created a clear need for remote health solutions to sustain health care practice, improve access to health care, and provide quality health care education.

Many aspects of health care and education were ready for XR and telehealth before the pandemic but remained underutilized due to equipment costs, unresponsive legislation, and limited health insurance coverage [98,99]. COVID-19 produced the conditions necessary to accelerate change, and now provides ample opportunity for those who embrace telehealth and complementary technologies. XR telehealth allowed providers to deliver services into patients’ own homes and naturalistic environments, which has long been a limitation of traditional clinical treatment [100]. Low-cost, off-the-shelf hardware and royalty-free software for therapy and rehabilitation made XR telehealth an economically feasible solution [101,102]. XR telehealth training and education also rose in response to COVID-19. The realistic, interactive capabilities of XR were broadly promoted as a solution to reach and educate patients and trainees over distances [103]. Simulations in VR and AR were common, safe, and repeatable alternatives to risky on-site in-person medical student training [104-106]. A cohort of medical-surgical students set to graduate during COVID-19 rated VR training as realistic for 77% of clinical assessments, 81% of treatment options, and 94% of diagnostics. After exposure to the virtual training, 84% of the cohort reported interest in the future use of VR for medical training and 90% overall satisfaction with virtual learning [107]. The rise of telehealth provided options for health care providers to sustain their practice when in-person visits became unfeasible. As part of telehealth, XR also proved to be a critical solution to provide health services and education amid pandemic conditions.

## XR Telehealth After COVID-19

### General Prospects

The impact of COVID-19 on the health care industry was sudden, severe, and broad. Longitudinal data are necessary to evaluate XR telehealth as an alternative to traditional in-person treatment and training. Nevertheless, XR telehealth served as a critical solution to the emergent conditions of COVID-19, maintenance of health care systems, and preparation of future providers. Telehealth is likely to become a staple of health care practice, as the majority of patients and more than 90% of providers intend to continue remote care beyond the resolution of COVID-19 [108-113]. Telehealth on its own is broadly effective and accepted but still leaves some patients and providers dissatisfied with their interactions with providers, specifically in their ability to feel present and build therapeutic relationships [114-116]. This lack of communicative nuance creates a vagueness in non-XR telehealth interactions that can be interpreted as awkward or even malicious [117]. Continued research and development of XR for telehealth can address some of these barriers to enhance therapeutic relationships, expand clinician capacity, and empower patients toward optimal health outcomes.

### XR Can Facilitate Telepresence to Strengthen Teletherapeutic Relationships

Therapeutic alliance is one of the best predictors of treatment success and health outcomes [118-120]. Therapeutic alliance is broadly defined by the relationship between the provider and patient, fostered through mutual agreement of clinical goals and the strategies to achieve those goals [121]. Non-XR telehealth is effective, accepted, and sustainable, but can make it difficult to replicate the communicative nuances and rapport building of in-person health care [122,123]. Health care providers who seem rigid, distant, or distracted (ie, typical attributes of non-XR telehealth [124,125]) produce poorer therapeutic alliances [126], which lead to higher chances of dropout, dissatisfaction, and negative health outcomes [127-129]. As a result, a small but important minority of providers believe that their patients do not enjoy telehealth as much as in-person care [116,130].

Preliminary evidence shows that XR can enhance remote interactions to strengthen therapist-patient relationships. Patients who received interactive CBT using VR avatars reported feeling less judged by their physical appearance, that the VR space was somewhere they could be honest and private with their therapist, and that the interaction felt more casual than an in-person clinic visit [56]. Likewise, physicians reported building rapid trust with their patients while jointly viewing patient body scans in VR and AR [131]. VR has also been promoted over non-VR alternatives for benefits such as more comfortable treatment, higher engagement, greater satisfaction, more consistent practice, greater skill transfer, and facilitation of nonverbal communication that improves therapist-client contact [132].

XR facilitates presence, when one perceives that the virtual environment is real [11]; embodiment, when one perceives a virtual body as one’s own real body [16]; and telepresence, when one perceives that they are inhabiting another place with virtual others [133]. Each of these aspects can aid in the establishment, improvement, and maintenance of therapeutic alliances in telehealth. Miloff and colleagues [134] developed an automated AR hologram embedded in VR exposure therapy and demonstrated that therapeutic alliance measures generalized to the virtual therapist. Although patients reported positive perceptions of this audio-only VR therapist, visually and behaviorally realistic VR therapists have been shown to evoke greater perceived presence [135]. Realistic XR avatars and XR interactions tend to evoke stronger physical and emotional closeness and greater confidence in the credibility of the therapist [135-137], which are key factors that influence an alliance with a virtual health care provider. However, XR telehealth is a nascent field, and more research is needed to understand how the two technologies interact to cultivate therapeutic alliances and impact health outcomes.

### XR With Telehealth Can Expand the Reach of Clinicians and Researchers

XR technologies were used frequently for health care education and training prior to COVID-19 [138-140], and this practice is expected to become increasingly common as traditional on-site medical education remains limited under the pandemic [86,104]. Simulation training in XR provides highly realistic experiences that deeply immerse learners in clinically realistic scenarios to facilitate skill acquisition and skill transfer for real application [141]. XR simulations can also provide repeatable exposure to important but improbable clinic scenarios, to prepare for states of emergency, and to access otherwise impossible views of medical procedure [103,142]. Remote XR simulations were used to facilitate skill development, prevent contagion spread, and rapidly disseminate COVID-relevant medical education during the pandemic [52,104,105]. Although the relationship between XR simulation training and clinical outcomes remains unclear [138,143-145], further exploration of remote XR training can help health care workers acquire, develop, and maintain cutting-edge skills with limited access to facilities or clinical populations. XR simulations stand to benefit from technologies to enhance realism and transfer of skills such as remote supervision, haptic feedback, anatomical replicas responsive to MR, and artificial intelligence to provide the most flexible and clinically beneficial education of future health care providers [146].

Health care is complex, fluctuating, and high-stakes work that often necessitates coordination of schedules, tasks, and information between multiple providers and teams. Unfortunately, hospitals are notoriously inefficient and error-prone due to a historic lack of human factors considerations in workflows, communications, equipment, user interfaces, and physical environments [147-150]. XR can play an important role in connected collaborative health care. Telesurgery with AR is a prominent example of how the marriage of XR and telehealth can improve health care work environments with surgeons receiving notes from expert consultants directly on their real-time view of the patient, seeing a proctor’s hands directing incisions, and delivering the expertise of medical specialists to regions with few or no local specialists [13,14,50,151]. The benefits of XR telesurgery have recently been demonstrated in nonsurgical medical teams for live distanced collaboration for inpatient unit care and coordination [51,152,153]. XR technologies enable immersive learning environments and pervasive sensor-display interfaces in the field. Telehealth enables real-time remote specialist consultation and expert supervision. The combination of XR and telehealth represents a system of potential force multipliers that can support, improve, and extend the capabilities of health care practitioners.

XR telehealth has increased patient access to health care, but this relationship has rich bidirectional potential to explore; clinicians and researchers can use XR telehealth to gain better access to patients and participants. Persky [154] described how controlled programmatic XR experiences could merge with remote clinical trials to minimize researcher and participant travel burdens; streamline and automate data collection; and critically improve engagement, retention, and procedural integrity. The recent popularity of consumer XR entertainment devices such as Facebook Oculus Quest 2, Sony PlayStation VR, and smartphone-based Google Cardboard can function as recruitment, enrollment, and data collection solutions with access to participants in their naturalistic settings. The use of fictionalized XR avatars to represent researcher and participant bodies can provide complete control over social manipulations and single- and double-blind study logistics [155]. Complicated data displays, technical instructions, and study processes such as informed consent are also easy for participants and researchers to visualize and interactively explore together in XR [156-158]. It will be critical to study XR for telehealth as a solution to extend historically localized research practices with mobility deployment to the general public and outreach for remote, underserved populations [159].

### XR Can Empower Patients to Seek Health Care and Improve Outcomes

Patients are empowered when they are treated as active collaborators in understanding and making decisions about their health care, rather than as passive subjects merely compliant with “doctors’ orders” [160]. Telehealth has already improved patients’ access to health care by removing geographical barriers (eg, travel costs and arrangements); however, remaining social and behavioral barriers to patient empowerment may be addressed with humanizing and engaging XR technologies.

There are widespread cultural stigmas that inhibit health-seeking behavior [161,162]. For example, men tend to avoid medical and mental health care to the point of early death and preventable decline in quality of life [163-165]. Other stigmas of diagnosis, gender, sexuality, ethnicity, body image, criminal history, and others similarly compromise health care utilization and outcomes [166-170]. Telehealth provides a beneficial distance that can make patients with stigmatized conditions feel confident and comfortable seeking services from their own homes [171-173]. XR can further enhance telehealth to include temporary, therapeutic distance from stigmatized bodies or identities. Avatars are 3D computer-generated models used in virtual environments to represent agents (eg, patients, providers, computer-controlled characters) [174]. The simulated nature of XR avatars makes them uniquely flexible for personalized health care approaches and interventions. One’s avatar can resemble their physical likeness in XR therapies faithful to what they would be like during an in-person health care visit [142]. Alternatively, patients may build a fictionalized avatar to provide a more comfortable degree of anonymity, extend embodiment-oriented therapies, and as a clinical enhancement for telehealth providers’ web-side manner [175,176]. Matsangidou and colleagues [56] recently demonstrated the many benefits of fictionalized avatars in VR treatment for both therapists and patients. Therapists tasked patients to build their own avatars, which provided useful clinical insights as to how the patient viewed themselves (ie, avatar appearance compared to real body). Patients attributed a wide variety of subjective benefits to the use of VR avatars, the most important of which were corroborated by therapists who noticed that avatars occasioned remarkable patient openness and trust. Interestingly, the therapists were also depicted with VR avatars in the form of simplistic cubes that were reported to enhance patients’ willingness to discuss difficult topics and engage in other mental health exercises. Telehealth allows patients to access health care with no need to travel, and XR can further enable access to care with no need for concern they will be judged. It will be important to explore avatars in XR telehealth as a solution for stigmas of visible medical conditions (eg, skin disease and burns) [177,178], criminal history or potential (eg, prevention of offense related to sexual preference) [179,180], and provide unprecedented opportunities for mental health and wellness [86,181,182].

The presence of and embodiment facilitated by avatars in responsive XR environments can result in simulated experiences that feel more real than physical reality (ie, hyperpresence) [183]. This hyperpresence may have tremendous clinical potential for remote health care. For example, patient motivation tends to be low for at-home rehabilitation due to the gap between unpleasant exercise and long-term health outcomes [184-186]. XR can boost the salience of physical rehabilitation with fictional but proactive feedback, similar to those in modern entertainment video games. Exaggerated body tracking in XR showed participants’ virtual bodies as stretching further and running faster than their real bodies, which significantly improved performance, enjoyment, and motivation for unsupervised rehabilitation-oriented exercises [187,188]. Hyperpresence in XR can also enable treatment contexts that in-person care and telehealth cannot. Traditional mental health treatments for internal stimuli (eg, emotional states or auditory hallucinations) rely on guided imagination that can alternatively be visualized and engaged with directly in XR [189,190]. Further, counterfactual hyperpresence can make health care more approachable for the shy or therapies that can be socially awkward. Group singing is an effective and cost-efficient intervention to promote respiratory health, but participants report lack of confidence when singing in front of others [191]. The same group-singing intervention in remote VR made participants feel socially uninhibited owing to their manifestation as anonymous and nonhuman VR avatars [54]. Hyperpresence is an emerging concept that merits investigation as a potential path for XR to enhance telehealth patient engagement, retention, and comfort. XR is currently used to alter patients’ sense of where they are and what they are doing but can also enhance patients’ sense of who they are in the future of telehealth [192].

## Remaining Barriers and Steps Forward

Telehealth revolutionized health care to meet patient needs at a distance. Although the majority of telehealth adoption was due to the pandemic, it is clear that telehealth will continue to expand beyond the resolution of COVID-19. We believe that XR is the next evolution for remote care built upon decades of foundational research and innovative demonstrations published in response to COVID-19. Toward that future, however, the barriers to XR are broadly similar to those of telehealth. Both technologies involve costly investments in equipment and training, can be abandoned after investment because of poor usability, and rely on broadband internet access that limits access on the basis of socioeconomic status and geographic location [151,193-195]. To realize the telepresent and empathetic future of XR telehealth, key barriers to mainstream adoption of both technologies must be addressed.

XR technologies involve complex electronic sensors, displays, and networks, which make costs a constant prohibitive factor. In 2000, a clinically sufficient VR headset with necessary computers and proprietary software could cost approximately US $17,000 (adjusted for 2021 inflation) [34]. However, high-end VR setups can be purchased today for use in one’s own home for about US $3000 total with a growing variety of free-to-use and open-source software [196]. Low-end XR (eg, Google Cardboard VR, Holokit AR) costs as little as US $15-$50, involves the use of smartphones many people already own, and has been applied with clinically significant treatment success [102,197]. Costs are anticipated to continue decreasing as consumer XR hardware becomes more established [198]. Concerns over costs can be further addressed with formal cost-benefit analyses of comparative treatment costs and impacts on quality of life.

Commercially produced XR hardware removes many barriers for health care providers interested in the technology, but this reliance on proprietary devices and software can be a double-edged sword for telehealth. Privacy is an ongoing concern with increasingly interconnected health care technologies [199]. This issue is particularly tricky with XR, as few devices exist on consumer markets that are compliant with regulatory health policies and the constantly evolving ways people use XR [40,200,201]. Although XR telehealth can feel private, the reality is that many XR devices and applications needlessly collect identifiable information and share user data with third parties. Certain XR devices such as Oculus Quest 2 are inoperable without data logging, and the manufacturer explicitly discourages its use with protected health information [202]. The recent rapid uptake of telehealth and XR continues to highlight the need for privacy and policy focused on health care end users [203]. Health care researchers, clinicians, consumers, and XR developers will need to organize and openly communicate to promote transparent and responsive privacy measures.

As a relatively new area of research, XR telehealth has a growing number of ethical concerns to address. First, the contexts in which XR telehealth are or are not appropriate have not been well established. XR telehealth may not benefit all health conditions or contexts equally. Treatment of high-risk conditions (eg, suicidality) still necessitates in-person responsiveness, while XR remains inaccessible to some (eg, those with chronic neurological conditions) or is unlikely to help others (eg, those with acute delirium). Second, practice competency is unclear with emerging telehealth and XR technologies. The broad foundational principles of competency are expected to be maintained as the public settles into widespread utilization of telehealth [204,205]. However, few telehealth practitioners are also experienced in software and hardware development. This current reality leaves most decisions about XR telehealth features and functions out of the hands of health care providers, making interdisciplinary collaborations a vital need into the future [206]. Third, the unique uses of XR for telehealth carry equal potential for misuse. Immersion, presence, copresence, and embodiment can facilitate remote health care, but it is not yet known how to best utilize these components or when one component should be emphasized over others. Modification of experiential states needs to be transparent and responsible in proportion to the potential risks [207]. Organizations for health care research and practice will need to establish and discuss ethical guidelines for XR telehealth. This is particularly important in light of reports that some are using nonmedical technology resources (eg, apps, games, websites) in lieu of qualified health care and the growing availability of self-help resources with little or no medical oversight [208,209].

In current and coming years, XR content offerings may be the greatest barrier for deployment via telehealth. There are currently about 60 million regular VR users and 91 million regular users of AR in the United States [210]. Major technology companies aim to make XR ubiquitous in the near future, which may make telehealth a more appealing use case for XR [211]. Although there is a growing variety of commercial XR telehealth options, the vast majority of XR consumption is for entertainment and industrial application [212]. XR for telemental health is promising, but uptake has been slow due to lack of usability or easy integration into existing clinical workflows [14,195,213]. It will be vital for researchers, clinicians, and developers to collaboratively assure that telehealth is a priority market in the design of XR hardware and software [214]. Furthermore, there are currently few sources of reputable, evidence-based, comprehensive information for telehealth providers to learn about and make treatment decisions with XR. Scholarly, clinical, and patient advocacy organizations should formally curate XR hardware and software to help navigate emerging treatment options for telehealth providers and patients.

The research literature on XR for telehealth is new, vast, and accelerating. The wide variety of XR hardware and software, study designs and populations, metrics and outcome variables, and vocabularies can be difficult to navigate and synthesize. The parameters of what constitutes VR, AR, and MR are still being explored, leaving overlap and obscurity in terms for practice and literature searches [215,216]. A consistent finding in XR narrative, systematic, and meta-analytic reviews is the variability in approaches that prevent formal comparison between studies [14,143,217]. Toward that end, Birckhead and colleagues [218] have provided recommendations to guide progressive and programmatic lines of XR research. Other good practices in this field of research include pretraining to orient participants to XR and minimize error, repeated exposure to detect and control for novelty confounds, and on-demand technical support during XR studies [54,219,220]. Failures with XR telehealth are equally important to publish as successes to accumulate details relevant to application and sustainability. Consistency of language, replicability, long-term effectiveness, and best practices for implementing XR telehealth must be disseminated to establish a comprehensive and conceptually systematic literature.

## Conclusion

Studies published during the COVID-19 pandemic showed that XR for telehealth helped health care providers stay safe during treatment of COVID-19 patients, improved the way health care was delivered to patients remotely, helped sustain a healthy frontline health care workforce, and supported the professional development of current and future providers. Toward the future of telehealth, we argue that XR can enhance interactive nuances and treatment options for telehealth patients, function as a force multiplier for health researchers and clinicians, and provide new options for at-risk patient populations. Cost, privacy, ethical practice, actionable practice guidelines, and improvements to research approaches must be addressed to fully realize the potential benefits of XR for telehealth. Despite these barriers, XR technologies have unique potential to enhance, extend, and expand the future of telehealth in and beyond the COVID-19 pandemic.

## Abbreviations

AR: augmented reality
CBT: cognitive behavioral therapy
MR: mixed reality
PPE: personal protective equipment
VR: virtual reality
XR: extended reality
